# Transitions in a genetic transcriptional regulatory system under Lévy motion

**DOI:** 10.1038/srep29274

**Published:** 2016-07-14

**Authors:** Yayun Zheng, Larissa Serdukova, Jinqiao Duan, Jürgen Kurths

**Affiliations:** 1 School of Mathematics and Statistics, Huazhong University of Science and Technology, Wuhan 430074, China; 2 Center for Mathematical Sciences, Huazhong University of Science and Technology, Wuhan 430074, China; 3 Department of Science and Technology, University of Cape Verde, Praia 7600, Cape Verde; 4 Department of Applied Mathematics, Illinois Institute of Technology,312-567-5335, Chicago 60616, USA; 5 Research Domain on Transdisciplinary Concepts and Methods, Potsdam Institute for Climate Impact Research, PO Box 60 12 03, 14412 Potsdam, Germany; 6 Department of Physics, Humboldt University of Berlin, Newtonstrate 15, 12489 Berlin, Germany

## Abstract

Based on a stochastic differential equation model for a single genetic regulatory system, we examine the dynamical effects of noisy fluctuations, arising in the synthesis reaction, on the evolution of the transcription factor activator in terms of its concentration. The fluctuations are modeled by Brownian motion and *α*-stable Lévy motion. Two deterministic quantities, the mean first exit time (MFET) and the first escape probability (FEP), are used to analyse the transitions from the low to high concentration states. A shorter MFET or higher FEP in the low concentration region facilitates such a transition. We have observed that higher noise intensities and larger jumps of the Lévy motion shortens the MFET and thus benefits transitions. The Lévy motion activates a transition from the low concentration region to the non-adjacent high concentration region, while Brownian motion can not induce this phenomenon. There are optimal proportions of Gaussian and non-Gaussian noises, which maximise the quantities MFET and FEP for each concentration, when the total sum of noise intensities are kept constant. Because a weaker stability indicates a higher transition probability, a new geometric concept is introduced to quantify the basin stability of the low concentration region, characterised by the escaping behaviour.

Regulation of gene expression plays a major role in molecular biological processes. The coexistence of multiple states have been found and analysed in a number of different genetic regulatory systems^1^,^2^,^3^,^4^,^5^,^6^. In gene expression and protein production, stochastic fluctuations are natural constituents of biochemical reactions. These fluctuations may induce a transient cellular differentiation of excitable gene regulatory circuits from a vegetative state to a competent state^1^,^2^, or lead to stabilisation of an unstable state^3^ and stochastic resonance^7^. Additionally, theoretical and experimental studies have established that appropriate random perturbations can induce a switch process between these states in the genetic regulatory models^7^,^8^,^9^,^10^,^11^,^12^, which is usually realized by manipulating a few key system parameters.

Previous investigations indicate that noise is a key factor for state transitions in genetic regulatory systems. These stochastic fluctuations have been mostly considered under the usual assumption of Gaussian distribution^7^,^8^,^9^. However, various biological systems evolve in random manners under non-Gaussian distribution, such as the foraging movement patterns of organisms, including albatrosses^13^ and many marine predators^14^, the behavior of the mutant individual population process^15^, or a protein-DNA target search model on seeking for binding sites in gene expression^16^,^17^.

Meanwhile, a large number of observations from biology experiments showed that the production of mRNA and proteins occur in a bursty, unpredictable, intermittent manner, which create variation or “noise” in individual cells or cell-to-cell interactions^18^,^19^,^20^,^21^,^22^,^23^,^24^. Such burst-like events result in a pulsatile fashion of high transcriptional activity followed by long periods of inactivity^20^,^21^,^22^ and exhibit heavy tailed distributions^20^. The characteristics of burst-like events, described above, appear to be appropriately modeled by the non-Gaussian Lévy motion but not by Brownian motion. Note that Lévy motions are sometimes called “Lévy flights” in physics^25^. A Lévy motion has a “heavy tail” distribution, as the tail estimate decays polynomially (unlike Brownian motion, whose tail estimate decays exponentially fast)^26^. It is recognised that at the microscopic level tiny jumps and short bursts may be regarded as the same phenomenon^27^. It has been also demonstrated that the noise intensity, the stability (Lévy) index of Lévy motion induces a switch process between distinct gene-expression states^11^. Moreover, It has been shown for a stochastic model that the average size of the bursts has an effect on the level of transcription factors^20^.

To examine the complex dynamical behaviour of a genetic regulatory system (Fig. 1a), Smolen *et al.*^4^ constructed a kinetic model (Eq. 2) described in Methods, which captures the salient features of autoregulation, signal-dependent phosphorylation of transcription factors (TFs), dimerization of transcription factors, crosstalk and feedback. A *transcription factor activator* (TF-A) is considered as part of a pathway mediating a cellular response to a stimulus. The TF-A activates transcription with a maximal rate *k*_*f*_ when they are phosphorylated, and forms a homodimer that can bind to specific responsive element DNA sequences (TF-REs). The tf-a gene incorporates one of these responsive elements, thus, the transcription increases. The regulatory activity of TFs is often modulated by phosphorylation. The TF-A monomer is degraded with a constant rate *k*_*d*_, and synthesized with a basal rate *R*_*bas*_. A constant *K*_*d*_ is regarded as the dissociation constant from TF-REs. The concentration of homodimer is proportional to the square of the TF-A monomer concentration, and we consider the binding processes to be evolving comparatively rapid.

Taking into account the biological significance and convenience of discussion, we choose appropriate parameters in this genetic regulatory system. The deterministic dynamical system (Eq. 2) has two stable states: *x*_−_, *x*_+_, and one unstable state *x*_*u*_, as indicated in Fig. 1b. However, the basal synthesis rate of the TF-A monomer *R*_*bas*_ is inevitably influenced by intrinsic random, molecular events, such as mutations and the transcription of mRNA or translation of proteins^8^,^20^. According to recent works^18^,^19^,^20^,^21^,^22^,^23^,^24^, these events exhibit burst-like behaviours in gene expression and thus it is appropriate to model them by Brownian motion and *α*-stable Lévy motion in equation (3) of Methods. The *α*-stable Lévy motion is a special type of Lévy motion defined by the stable Lévy random variables. In present paper, we regards *α*-stable Lévy motion as a pure jump motion, which has larger jumps with lower jump probabilities for values of *α* close to 0, while it has smaller jumps with higher frequencies for large values of *α* (1 < *α* <2).

Noise-induced genetic transitions of states can be treated as a first exit problem. Two concepts were applied to the exit phenomenon: *mean first exit time* (MFET) and *first escape probability* (FEP). According to the theory of large deviation^28^,^29^,^30^, the height of the potential barrier plays a pivotal role in the discussion of the first exit problem in the Gaussian case. That is the reason why it was thought that a deeper well would correspond to a more stable state. When a system is driven by (non-Gaussian) *α*-stable Lévy motion, it was shown that the MFET increases with a power *ε*^−*α*^ of the noise intensity *ε*^31^,^32^,^33^, instead of an exponential law in the case of Brownian motion. The asymptotic estimate of the MFET and the FEP were found when *ε* is sufficiently small^32^,^33^,^34^,^35^,^36^. The nonlocal elliptic partial differential equations have been considered to solve these two quantities^26^. Recently, authors have also developed efficient and accurate numerical schemes to simulate exit problems from the corresponding nonlocal partial differential equations^37^,^38^,^39^.

From a genetic regulatory point of view, biologists have focused on primarily on the high end of the TF-A monomer concentration corresponding to the high degree of transcriptional activity. Moreover, experimental observations have indicated that adjusting the concentration of transcriptional activators affects the overall level of transcription^19^,^20^,^22^,^23^. If we regard the low concentration state as the “off” state and the high concentration state as the “on” state, the transition for the TF-A monomer concentration from the low to the high concentration state corresponds to a switch process. Such transitions might explain how a brief pulse of hormone or neurotransmitter could elicit a long-lasting cellular response^4^. Therefore, we only focus on how the TF-A monomer concentration shifts from the low to the high concentration state in this paper.

We assume that the TF-A monomer concentration switches to high concentration state if it exits from right boundary of *D* = (0, *x*_*u*_). A simulation analysis is conducted to investigate the relative importance of Gaussian and non-Gaussian noises in affecting behaviors of the TF-A monomer concentration model, by analyzing two deterministic quantities: the mean residence time or mean first exit time (MFET) in *D*, and the first probability of escape (FEP) to the high concentration state from *D*. A shorter MFET or higher FEP in the low concentration region facilitates a transition. Moreover, because lower stability indicates higher probability for transition, we will define a new concept (called *stochastic basin of attraction*) to quantify this aspect of the stability of the low concentration region. These tools and related findings will provide new insights into natural mechanisms of transitions between low and high concentration states in a genetic regulatory system.

Our research is mainly motivated, on the one hand, by the lack of studies in the gene transcriptional regulatory system under non-Gaussian Lévy fluctuations, and on the other hand, by the limitation of techniques used in the existing studies. The existing techniques have been applied to various experimental observations^1^,^2^,^18^,^19^,^20^ or by typically assuming that the underlying distribution is stationary^7^,^8^,^11^. However, the available experimental data are limited to certain specific parameters, and not all systems have a stationary distribution. In the present work, we will fill this gap and employ tools of stochastic dynamical systems^26^. The advantage of this approach is that we study the dynamical behaviors of the TF-A monomer concentration under Lévy fluctuations by two deterministic quantities: the MFET and the FEP. We compute these quantities by numerically solving two deterministic nonlocal or differential-integral equations (Eqs 6 and 9), corresponding to the stochastic dynamical model (Eq. 3), without conducting sample-wise or Monte Carlo simulations.

## Results

**Figure 1:**
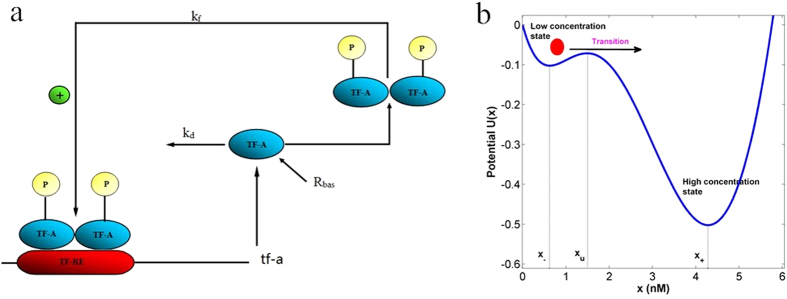
Model of genetic regulation with a positive autoregulatory feedback loop. (**a**) The transcription factor activator (TF-A) activates transcription with a maximal rate *k*_*f*_ when phosphorylated (P) and binds as a dimer to specific responsive-element DNA sequences(TF-REs). TF-A is decomposed with rate *k*_*d*_ and synthesized with rate *R*_*bas*_. (**b**) The bistable potential. The parameter values are *k*_*f*_ = 7.5 *min*^−1^, *K*_*d*_ = 10, *k*_*d*_ = 1 *min*^−1^, and *R*_*bas*_ = 0.1 *min*^−1^. The stable states are *x*_−_ ≈ 0.1089 *nM* and *x*_+_ ≈ 5.9467 *nM*, and the unstable steady state is *x*_*u*_ ≈ 1.5445 *nM*.

For our numerical experiments, we fix the regulatory system parameters *k*_*f*_ = 6, *K*_*d*_ = 10, *k*_*d*_ = 1, and *R*_*bas*_ = 0.4 as suggested in ref. 8. Thus, the deterministic genetic regulatory dynamical system (Eq. 2) exhibits the two stable states at *x*_−_ ≈ 0.62685 (the low concentration stable state), *x*_+_ ≈ 4.28343 (the high concentration stable state), and the unstable state at *x*_*u*_ ≈ 1.48971. The low concentration region *D* = (0, 1.48971) encloses the low concentration stable state *x*_−_ ≈ 0.62685 (Fig. 1b). We now focus on the impact of Gaussian Brownian motion and non-Gaussian *α*-stable Lévy motion on the mean first exit time, first escape probability and basin of attraction in TF-A monomer concentration model.

**Figure 2:**
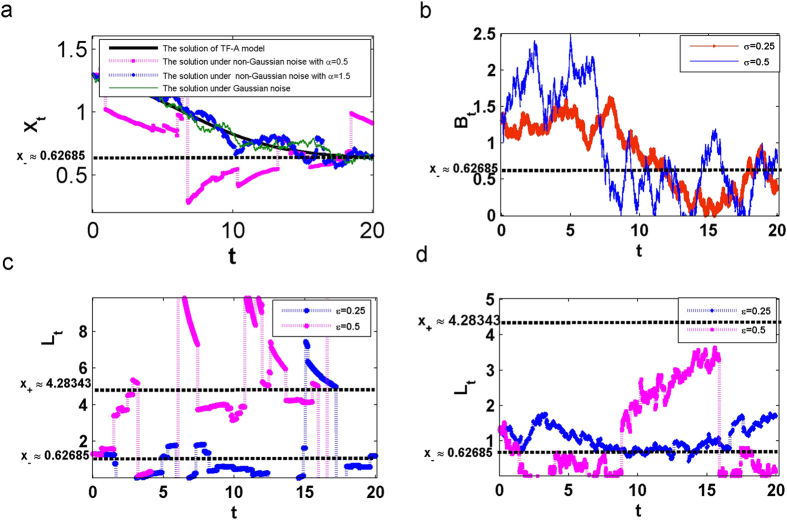
Effect of noise on the solution trajectories of the stochastic model (Eq. 3) with initial concentration *x*_0_ = 1.3. (**a**) Solutions are driven under no noise, Gaussian noise, and non-Gaussian noise with *α* = 0.5, 1.5, for noise intensity *σ* = *ε* = 0.05. (**b**) Noise intensities *σ* for (Gaussian) Brownian motion. (**c**) Noise intensities *ε* for (non-Gaussian) *α*-stable Lévy motion with *α* = 0.5. (**d**) Same as (**c**) except *α* = 1.5.

We can look at dependence of the TF-A monomer concentration on the noise intensity and the size of jumps. In the deterministic model (Eq. 2), for the initial concentration *x* _0_ = 1.3 < *x*_*u*_, the TF-A monomer concentration *x*(*t*) (black solid line in Fig. 2a) will approach to the low concentration stable state *x*_−_ as *t* goes to infinity. While, in the stochastic model (Eq. 3), the stochastic path (trajectory) *X*_*t*_ in the case of Brownian motion does not deviate much from the deterministic path, and the paths are continuous but almost surely nowhere differentiable. However, in the case of *α*-stable Lévy motion, a stochastic path has infinitely many small jumps for the Lévy motion index *α* = 1.5, while it has larger jumps with lower jump frequencies for small values of *α* = 0.5, and the trajectory for *α* = 0.5 is easier to switch to the high concentration stable state *x*_+_ than for *α* = 1.5. Whatever Gaussian and non-Gaussian noise, increasing the noise intensity parameters *σ* and *ε* cause larger perturbations on the trajectory as shown in Fig. 2. This implies that the frequency and magnitude of these two types of noise substantially affect the TF-A monomer concentration within a cell in gene expression.

### Shorter MFET for higher noise intensity

Now, we discuss separately the impact of the Brownian motion and *α*-stable Lévy motion on the MFET, which is the mean time of the TF-A monomer concentration *X*_*t*_ that remains in the low concentration region *D* = (0, 1.48971) before exiting to the high concentration state. The MFET *u*(*x*) is described by the differential-integral equations in equation (6), which is in detail described in the Methods. Notice that it has a nonlocal “Dirichlet” condition, i.e., the value of *u*(*x*) equal to 0 for the entire exterior domain *D*^*c*^ (the complement set of *D*). In particular, the boundary values are *u*(0) = *u*(1.48971) = 0.

**Figure 3:**
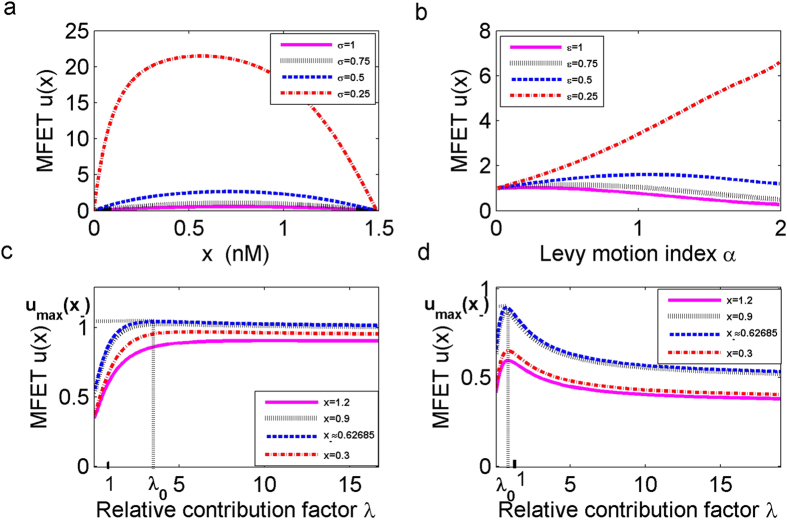
Effect of noise intensity on the mean first exit time (MFET) *u*(*x*) with *D* = (0, 1.48971). (**a**) Effect of Gaussian noise intensity *σ* on the MFET. (**b**) Effect of Lévy motion index *α* and noise intensity *ε* on the MFET at *x*_−_ ≈ 0.62685. (**c**) Combined effect of Gaussian and non-Gaussian noises on the MFET for different initial concentrations *x* = 0.3, 0.9, 1.2 and *x*_−_ ≈ 0.62685 with *α* = 0.5. (**d**) Same as (**c**) except *α* = 1.5.

In the deterministic model (Eq. 2), the trajectories stay in the low concentration region *D* forever. When the reaction rates of synthesis is subject to a stochastic perturbations defined in equation (3), the residence time in this region becomes finite. It demonstrates that the longest residence time corresponds to the concentrations near the *x*_−_ ≈ 0.62685, since the potential energy is smallest at there. Another observation from Fig. 3a,b are that larger values of noise intensities *σ* and *ε* lead to a shorter residence time. Besides, compared with Brownian motion, the Lévy motion shortens the MFET when the value of noise intensities *σ* and *ε* are fixed.

### Shorter MFET for higher Lévy noise intensity and larger jumps

In Fig. 3b, analyzing the influence of the *α*-stable Lévy motion parameters, the index *α* ∈ (0, 2) and the noise intensity *ε* ∈ [0, 1], on the MFET for the low concentration stable state *x*_−_, we conclude that the MFET increases with increasing *α*, when *ε* is small. This indicates that the state *x*_−_ becomes more stable. Also, we find that for sufficiently large values of *ε*, there are critical values of *α* leading to change of the MFET from increasing to decreasing, which is in accordance with the stability behaviour of the state *x*_−_.

The results indicate that we have to consider a higher *ε* and a smaller *α* (larger jumps with lower jump frequencies) if we expect to reduce the MFET. Thus, the TF-A monomer concentration *X*_*t*_ is easier to shift to the high concentration state in this situation. This result agrees with the observation in ref. 20. The preceding result implies that a switch process can be induced by varying noise intensities *σ*, *ε* and the Lévy motion index *α* in the genetic regulatory system. This is also realized by manipulations of *k*_*f*_ in existing works^4^.

### Optimal Gaussian and non-Gaussian noise intensities for maximizing MFET

Having discussed the individual impact of Brownian motion or *α*-stable Lévy motion on the MFET, we examine now the combined effect of these two types of noise. The important question is which one has a greater contribution to the residence time and how the input of the noises make the MFET the largest. We define a parameter *λ* as the *relative contribution factor* (RCF), which is the ratio of relative intensity between the non-Gaussian noise intensity *ε* and the Gaussian noise intensity *σ* in stochastic model (Eq. 3), i.e.,


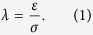


We take *σ* to be within (0, 1]. We assume that *ε* + *σ* = 1 to guarantee that the total quantity of noises are determined. If *λ* ≤ 1, it implies that the Brownian motion has a major role on the maximum residence time, but if *λ* > 1, then the *α*-stable Lévy motion has a greater impact than the Brownian motion on the MFET.

Considering the impact of *λ* on the MFET in the range *D* of the low concentration, four different initial concentrations, i.e., *x* = 0.3, 0.9, 1.2 and *x*_−_ ≈ 0.62685, are selected. Fig. 3c,d show that the MFET is increasing, as *λ* increases, up to the maximum value of the MFET, and then, further increase of *λ* leads to shorter mean residence times. When *λ* reaches a certain threshold value *λ*_0_, we observe that the MFET remains nearly constant with increasing *λ* for different levels of concentration.

In details, for a small value of *α* (0 < *α* < 1), such as *α* = 0.5 (Fig. 3c), the concentration of the TF-A monomer focuses on the low concentration stable state *x*_−_. The maximum value of the MFET corresponds to the threshold *λ*_0_ > 1, i.e., the intensity of the non-Gaussian noise is *λ*_0_ times larger than the Gaussian noise. This suggests that the *α*-stable Lévy motion plays a crucial role in the residence time of the TF-A monomer concentration for the larger jumps. Besides, as *λ* is increasing, keeping other factors fixed, the MFET is decreasing a bit at first, and then remains identical. For a larger value of *α*, such as *α* = 1.5 (Fig. 3d), the threshold *λ*_0_ < 1 due to the significant effect of the Brownian motion, and the value of MFET presents a greater decrease before entering into a constant level.

These results imply that we find the optimal proportion of Gaussian and non-Gaussian noise which has a desirable effect on the MFET. This optimal proportion is the threshold *λ*_0_. For each TF-A monomer initial concentration *x*, there exists an optimal proportion for these two noises, for which the MFET of the TF-A monomer concentration becomes the largest, indicating the greater stability of the low concentration state. The MFET becomes shorter or remains a nearly constant level when the value of *λ* is changed, corresponding to decreasing or increasing *ε* and *σ*.

### Two scenarios of escaping

Now, let the starting concentration *x* be in the low concentration state *D*, we quantify the impact of noise on the probability of first switching to the high concentration state *E*, via first escape probability (FEP). The FEP *p*(*x*) is a solution of the differential-integral equations in equation (9), which is presented in Methods. We assume that the probability is 1 when the stochastic path (trajectory) *X*_*t*_ lands in a subset *E* of *D*^*c*^ (the complement of *D*), otherwise the probability is 0, which are described by the Balayage-Dirichlet exterior boundary condition. The high escape probability *p*(*x*) value indicates the high likelihood for the TF-A monomer concentration *X*_*t*_ transition from the low to high concentration state. Considering the escaping phenomenon, we have to distinguish two situations: if the escape region *D* is adjacent to the escape-target region *E* (Scenario 1) or not (Scenario 2),

- Scenario 1: The starting concentration of the TF-A monomer belongs to *D* = (0, *x*_*u*_), and the probability exits to outside of *D* from the right boundary *x*_*u*_ reaching *E* = [*x*_*u*_, +∞) with the boundary condition *p*(*x*_*u*_) = 1.
- Scenario 2: The concentration of the TF-A monomer *X*_*t*_ lands in the specific local high concentration region *E* = [*c*, *d*], which is not adjacent to *D* = (0, *x*_*u*_). The probability is equal to 0 when the *X*_*t*_ lands in the outside of *D* except *E* (i.e., *D*^*c*^\*E*) with the boundary condition *p*(0) = *p*(*x*_*u*_) = 0.

### Effect of Lévy motion index and noise intensity on the FEP for different concentrations in Scenario 1

**Figure 4:**
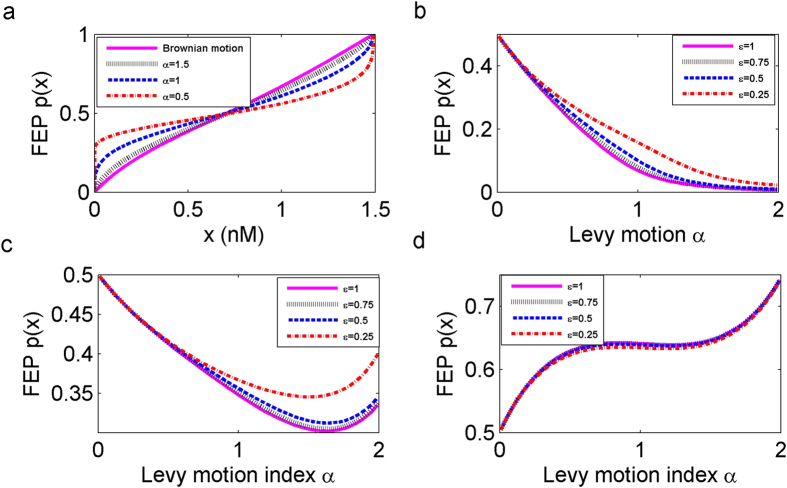
First escape probability (FEP) *p*(*x*) from the low concentration region *D* = (0, 1.48971) to the adjacent high concentration region *E* = [1.48971, +∞). (**a**) The FEP *p*(*x*) for *α*-stable Lévy motion with *α* = 0.5, 1, 1.5 and Brownian motion, for noise intensities *σ* = *ε* = 0.5. (**b**) Effect of Lévy motion index *α* and noise intensity *ε* on the FEP at *x* = 0.012. (**c**) Same as (**b**) except *x*_−_ ≈ 0.62685. (**d**) Same as (**b**) except *x* = 1.4.

We are concerned with how to enhance the likelihood for state transition from the low to high concentration. As seen in Fig. 4a, an intersection point appears near the *x*_−_ ≈ 0.62685. When the starting concentrations *x* is larger than the intersection point, the probability of escaping is larger as the value of *α* increases. Moreover, at a fixed initial concentration *x*, the escape probability *p*(*x*) is the largest in the case of Gaussian noise (*α* = 2). The behaviour of the escape probability is opposite when the starting concentration *x* is smaller than the intersection point.

In addition, we investigate effects of Lévy motion parameters *α* and *ε* on the FEP for the following three representative the TF-A monomer initial concentrations *x* = 0.012, 1.4 and *x*_−_ ≈ 0.62685. As *α* becomes larger, for the different values of the noise intensity *ε* =  0.25, 0.5, 0.75,1, the FEP tends to decrease for *x* = 0.012 as shown in Fig. 4b. While, in Fig. 4d, we notice that the FEP increases as *α* increases for *x* = 1.2. The rate of increase is smaller than the decrease rate in these two figures. For the initial concentration at the stable state *x*_−_ ≈ 0.62685 in Fig. 4c, the behavior of the escape probability does not change monotonically for each *ε*, instead, the FEP first decreases, reaches a minimum, and then increases with increasing *α*. According to these results, the behaviour of the transition probability depends on the concentrations of the TF-A monomer. Besides, the bigger *ε* has more evident effects on the FEP with increasing *α*.

### Larger jumps in α-stable Lévy motion promote the transition in Scenario 2

**Figure 5:**
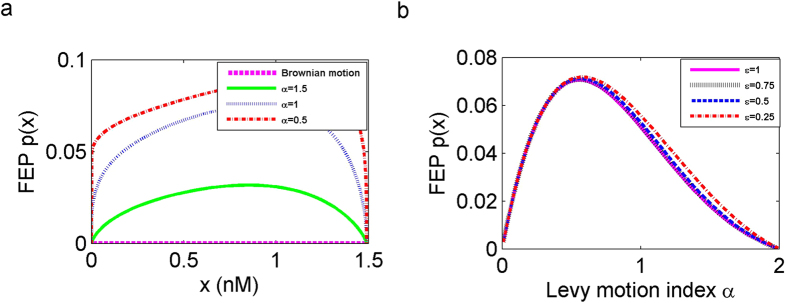
First escape probability (FEP) *p*(*x*) from the low concentration region *D* = (0, 1.48971) to a non-adjacent high concentration region *E* = [3, 5]. (**a**) The FEP *p*(*x*) for *α*-stable Lévy motion with *α* = 0.5, 1, 1.5 and Brownian motion, for noise intensities *σ* = *ε* = 0.5. (**b**) Effect of Lévy motion index *α* and noise intensity *ε* on the FEP at *x*_−_ ≈ 0.62685.

Next, we look at the effect on the FEP when a selected target domain *E* = [3, 5] is not adjacent to *D* = (0, 1.48971). In Fig. 5a, we find that there is no intersection point, and the value of FEP *p*(*x*) is smaller comparing with Scenario 1 (Fig. 4a), for the same starting concentration *x*. Because of the distance between *D* and *E* becomes farther, this result is consistent with the existing theories^32^,^33^. The escape probability profile is flat in the middle of the domain and drops to zero quickly near both boundaries, determined by the boundary conditions (*p*(0) = *p*(1.48971) = 0) in Scenario 2. The FEP becomes bigger with a decrease of *α*, i.e., larger jumps with lower frequencies are more beneficial for the long distance range shifting. The probability is zero for Brownian motion, because the trajectory of the Brownian motion is continuous, for almost all samples unlikeness trajectory of the Lévy motion, which has right continuous with left limit paths, with countable jumps in time. Thus, Brownian motion with continuous paths can not jump to *E* which is not adjacent to *D*. It indicates that Gaussian fluctuations can not induce transition phenomena by itself in Scenario 2. Figure 5b shows that as *α* increases, the FEP for the starting concentration at *x*_−_ ≈ 0.62685 tends to increase to its maximum value and then decreases, and higher values of *ε* have a greater effect on decreasing FEP. The behaviour of FEP for the other starting concentrations in *D* are similar to this case .

### In Scenario 1, both Brownian motion and α-stable Lévy motion advance transition in different ways

We would like to further investigate the transition probability for the *α*-stable Lévy motion combined with Brownian motion. The measurement parameter *λ* in equation (1) indicates the relative intensity of these noises in the overall impact of perturbations.

**Figure 6:**
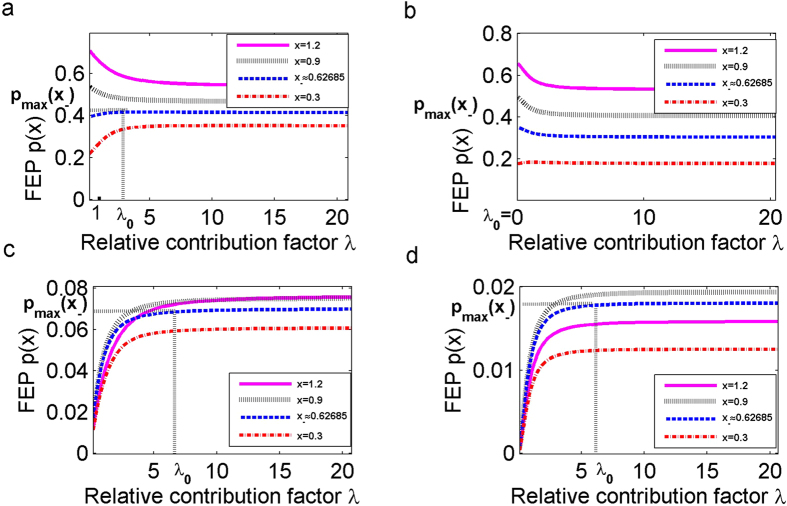
Effect of the relative contribution factor *λ* on first escape probability (FEP) *p*(*x*). (**a**) The escape probability *p*(*x*) for Scenario 1 with *α* = 0.5. (**b**) Same as (**a**) except *α* = 1.5. (**c**) The escape probability *p*(*x*) for Scenario 2 with *α* = 0.5. (**d**) Same as (**c**) except *α* = 1.5.

In the first Scenario, for small value of *α* = 0.5 and the lower initial concentrations of the TF-A monomer, such as *x* = 0.3, and *x* ≈ 0.62685, Fig. 6a shows that increasing *λ* causes increasing the FEP, and the value of FEP remains constant when *λ* reaches the threshold *λ*_0_ > 1 for the low concentration stable state *x*_−_. This *λ*_0_ indicates that the *α*-stable Lévy motion promote the transition with increasing *λ*. For higher starting concentrations *x* = 0.9, 1.2, the FEP is the largest for the threshold *λ*_0_ = 0, which implies that the synthesis reaction rate is only affected by the Brownian motion. As *λ* increases, the input of the *α*-stable Lévy motion component inhibits at first, and then it has no further effect on the probability of the transition. Figure 6b shows that the behaviour of the escaping probability is in agreement with the corresponding result in *α* = 0.5, for three values of initial concentration, i.e *x* = 0.3, 0.9,1.2. However, for the starting concentration at *x*_−_, the non-Gaussian noise does not promote but reduces the probability of escape with increasing *λ*. It indicates that the behaviour of the optimal proportion *λ* is also affected by the Lévy motion index *α* even for the same concentration.

We conclude that there is an optimal ratio of the influence of the Brownian motion and *α*-stable Lévy motion on the FEP for all concentrations in Scenario 1. For lower concentrations, the pure jump motion induces a greater contribution to shift to high concentration state, improving the efficiency of the transcription. While for higher concentrations, the impact of Brownian motion on the transition probability becomes stronger in the genetic regulatory system.

### In Scenario 2, *α*-stable Lévy motion is more likely to induce a transition

Next, we assume that the regions *D* = (0, 1.48971) and *E* = [3, 5] are not adjacent. Figure 6c,d illustrate that the increase of *λ* leads to the largest probability, corresponding to thresholds *λ*_0_ > 1, then the FEP will keep nearly flat. With the increase of *λ*, the FEP also undergoes a sudden increase up to a certain level, starting from which the FEP takes a near-constant value. Anther observation is that the FEP is higher for *α* = 0.5 than for *α* = 1.5 with the same concentration. It means that it is easier to accomplish a transition with *α* closer to 0 in Scenario 2, which relates to larger jumps with lower jump probabilities. When *λ* = 0, it means that the system is only under the influence of Brownian noise, the probability of escape to the high concentration state goes to zero, as seen in Fig. 5a. The preceding result shows that the *α*-stable Lévy motion has a larger contribution to the transition than the Brownian motion in Scenario 2.

### Definition of stochastic basin of attraction

The preceding results show that the low concentration state is less “stable” when the MFET is shorter or the FEP is higher, and then it is easier to switch to the high concentration state for the TF-A monomer concentration *X*_*t*_. This is beneficial for transcriptional activities. Thus, it is interesting and relevant to investigate the stability of the low concentration state or low concentration region. Menck *et al.*^40^ introduced basin stability, a new concept of stability, that is measured by the volume of basin of attraction. The basin of attraction is a property of deterministic dynamical systems. It is well known that the basin of attraction of an invariant set is the set of all initial states for which the solution of the system converges to this set^41^. We will extend this to a new concept: *stochastic basin of attraction* (SBA) for stochastic dynamical systems. It will be used to quantify the basin stability based on the basin’s volume. The volume is here simply regards as the interval length of the basin of attraction in 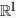. A concrete approach based on the MFET *u*(*x*), and the FEP *p*(*x*) is utilized to quantify the SBA. We now formally define the SBA for a closed set *K*.

*The stochastic basin of the closed set K is denoted by* 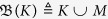
*. It is the collection of all initial points* 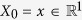 *satisfying: M* = {*x* ∈ *K*^*c*^|*p*(*x*) > p^*^}*. Here p*(*x*) *is the probability for all initial points return to the closed set K from outside of K. The closed set K can be quantified by the MFET u*(*x*)*, K* = {*x* ∈ *B*|*u*(*x*) ≥ u^*^}*, i.e., the stochastic solution X*_*t*_ *starting in K and remains there for a finite time and B is the deterministic basin of attraction of a invariant set. The closed set K can be taken as an invariant set in the sense of stochastic dynamics.*

In other words, SBA is the set of all initial concentrations whose trajectories have a high probability to return to the set where the stochastic solutions may remain for a long (but finite) time. Going back to the TF-A monomer concentration deterministic model, the low concentration stable state *x*_−_ is an invariant set, the deterministic basin of attraction *B*(*x*_−_) = [0, 1.48971], where the TF-A monomer concentration *x*_*u*_ ≈ 1.48971 is an unstable state. We focus on discussing the size of the basin of a closed set under Gaussian and non-Gaussian noises. For convenience, we assume that all the escaping domains are bounded. The detailed descriptions and simulation results are presented below.

### Stochastic basin of attraction under Brownian motion

**Figure 7:**
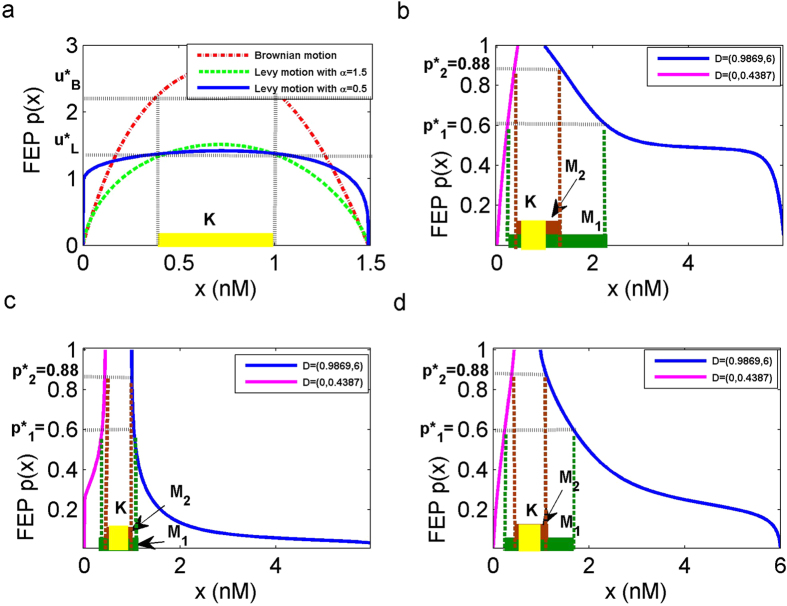
Effect of noises on the stochastic basin of attraction. (**a**) The closed set *K* with noise intensities *σ* = *ε* = 0.5, exit domain *B*(*x*_−_) = (0, 1.48971). (**b**) The stochastic basin of attraction 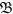 in the model with Brownian motion, return to *K* from domain *D* = (0, 0.4387)∪(0.9869, 6). (**c**) Same as (**b**) except in the model under *α*-stable Lévy motion, with *α* = 0.5. (**d**) Same as (**c**) except *α* = 1.5.

Considering that the size of the basin of attraction was effected by the Gaussian noise (i.e., *ε* = 0) at first. Figure 7a illustrates that the mean residence time is longer than 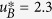 when the TF-A monomer initial concentration *x* belongs to the closed set *K* = [0.4387, 0.9869] which is marked yellow. It indicates that these initial concentrations start in *K* and remain there for a longer time than other concentrations. Next, we expect to estimate which initial concentrations *x* will return to *K* when the rate of synthesis is disturbed by Brownian motion. In Fig. 7b, the probability of escaping to *K* is up to 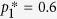 as the initial concentration of the TF-A monomer *x* is close to 0.222 and 2.295, i.e., the part of the low concentration *x* ∈ [0.222, 0.4387] will return to *K*, and the high concentration *x* ∈ [0.9869, 2.295] will also be switched to *K* under Gaussian noise. These sets by definition are parts of the set *M*_1_ marked green. Thus, the stochastic basin of *K* is the 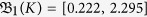 (the interval length of two green dotted lines) with probability 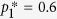. This implies that the initial concentration of the TF-A monomer *x* in *M*_1_ has 60 percent possibility return to *K* and remains there. In conclusion, a feasible method is provided here to measure the basin of attraction under stochastic influences.

When we choose a larger transition probability, such as 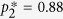, the set of escaping concentrations becomes *M*_2_ = [0.3685, 0.4387]∪[0.9869, 1.358] which is marked brown. The SBA of the closed set *K* is 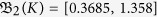 (the interval length of two brown dotted lines). Thus, the concentration of the TF-A monomer that focuses on *M*_2_ has a higher probability return to *K*, while the size of the escape region *M*_2_ (brown) is smaller than *M*_1_ (green). Therefore, the size of the basin 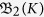 is also smaller than the previous one 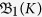. It declares that the size of basin 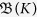 depends on the transition probability *p*(*x*) when *K* is fixed.

### Stochastic basin of attraction under *α*-stable Lévy motion

Now we use the preceding method to further investigate the size of the SBA evolution under *α*-stable Lévy motion (i.e., *σ* = 0). We compare the size of the stochastic basin of *K* for *α* = 0.5, 1.5. The closed set *K* = [0.4387, 0.9869] has the same length as in case of Brownian motion with the residence time 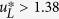. For the small value *α* = 0.5 (Fig. 7c), the TF-A monomer initial concentrations *x* are shifted to *K* with probability 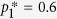 from the green region, which tends to be relatively smaller. In contrast, for the larger value *α* = 1.5 (Fig. 7d), the length of green region becomes bigger with the same escape probability, i.e., more concentrations return to *K* with a bigger value of *α*, corresponding to smaller jumps with higher frequencies. Consequently, the size of stochastic basin of *K* becomes bigger for *α* = 1.5. The results for the larger escape probability, such as 0.88, are in accordance with the results described above. Besides, like in the case of Brownian noise, the larger probability return to *K* is associated with a smaller size of the basin of attraction, and the same results are also manifested in the case of *α*-stable Lévy motion.

The results on the SBA show that the size of basin of *K* becomes bigger for *α* values closer to 2, especially for Brownian motion (corresponding to *α* = 2). As in discussed^40^, the size of stochastic basin of attraction for *K* expands due to Lévy fluctuations for smaller jumps with higher jump probabilities. This indicates that the low concentration region *K* becomes more stable (in the sense of basin stability) when the fluctuations are of Lévy type with larger *α*, and hence is less in favor of transcription.

## Discussion

In summary, we have examined the impact of (Gaussian) Brownian and (non-Gaussian) *α*-stable Lévy environmental fluctuations on the synthesis reaction rate of a specific TF-A monomer concentration dynamical model (i.e., a stochastic differential equation). The mean first exit time (MFET) and the first escape probability (FEP) are computed to quantify the mean residence time in the low concentration region before exit to the high concentration state and the probability that the TF-A monomer concentration shifts from the low concentration state to the high concentration one.

Using both analytical and numerical tools, we have found that the noise intensity *σ*, *ε*, the jump frequency and the jump size of *α*-stable Lévy motion have significant and delicate influences on the MFET and the FEP. There are optimal proportions of Gaussian and non-Gaussian noise intensities that maximize these two quantities, when the total sum of noise intensities are kept fixed. Beyond this optimal combination, the increases of the relative contribution factor *λ* does not further affect the MFET and the FEP.

Due to the competition between the Lévy motion index *α* and the noise intensity *ε*, the behaviour of the FEP depends on the concentration of the TF-A monomer in Scenario 1 (when the low concentration region and the high concentration region are adjacent), the transition probability increases as the TF-A monomer initial concentration *x* takes values near the high concentration region. In Scenario 2 (when the low concentration region and the high concentration region are non adjacent), Brownian fluctuations in the stochastic TF-A monomer concentration model can not induce switching phenomena by itself, since the solution paths are continuous and thus can not jump. This is unlike the stochastic TF-A monomer concentration model with *α*-stable Lévy motion. However, Brownian motion has an effect, although hinders rather than promotes transition, on the switching when combined with *α*-stable Lévy motion.

We have also presented a method to measure the size of the stochastic basin of attraction for a closed set (taken as a set containing the low concentration state) in the TF-A monomer concentration stochastic model. This enables us to quantify the basin stability of the low concentration state. For this specific stochastic model, we have observed that a smaller size of the SBA implies a higher probability return to the closed set *K*. Moreover, the low concentration region *K* becomes more stable when the fluctuations are of Lévy type with larger *α*, and hence is less in favor of transcription.

## Methods

### The TF-A monomer concentration deterministic model

To investigate effects of noises on the transition to high concentration state from the low concentration one, we presented two classes of mathematical models. First of all, we consider the kinetic transcriptional factor activator (TF-A) model of a single genetic regulatory system with a positive autoregulatory feedback loop^4^. The TF-A monomer regulates transcription with a maximal phosphorylated rate *k*_*f*_ and binds to specific responsive-element DNA sequences (TF-REs). The TF-A monomer is decomposed with rate *k*_*d*_ and synthesized with rate *R*_*bas*_. These simplifications give a model with a single ordinary differential equation for the TF-A monomer concentration *x*


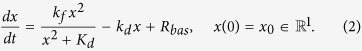


The equation (2) can be written as 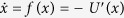 with the potential energy is


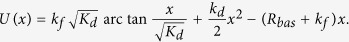


Under the following condition on the parameters


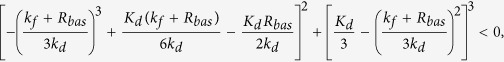


this deterministic system has two stable concentration states 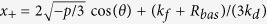, 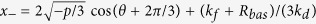 and one unstable state 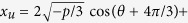 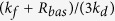. Here 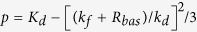, 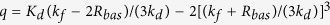, and 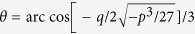.

### The TF-A monomer concentration stochastic model

Secondly, we examine the dynamical effects of noisy fluctuations, on the evolution of the TF-A in terms of its concentration, involves both Gaussian and non-Gaussian distributions. In the introduction, we have discussed about non-Gaussian characteristics of fluctuation in transcriptional from gene. To represent the reaction rate of synthesis of noises fluctuations, our model is written as a stochastic differential equation


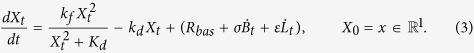


Here 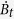, 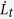 are Gaussian and non-Gaussian noise, which can be modeled by a scalar standard Brownian motion, and a scalar *α*-stable Lévy motion with the generating triplet (0, 0, *ν*_*α*_). In this paper, this *α*-stable Lévy motion is a pure jump process and independent of *B*_*t*_. We define a parameter *λ* as relative contribution factor, which is the ratio of relative intensity between the Gaussian noise and the non-Gaussian noise, i.e., *λ* = (*ε*)/(*σ*), *σ* belongs to (0, 1]. We assume that *ε* + *σ* = 1 to guarantee the total quantity of noises intensity are determined.

### The *α*-stable Lévy motion

The Lévy motion (process) *L*(*t*) is thought to be an appropriate model for non-Gaussian processes with jumps, which has properties of stationary and independent increments. That is, for any *s*, *t* with 0 ≤ *s* ≤ *t*, the distribution of *L*(*t*) − *L*(*s*) only depends on *t* − *s*, and for any partition 0 = *t*_0_ < *t*_1_ < … < *t*_*n*_ = *t*, *L*(*t*_*i*_) − *L*(*t*_*i*−1_), *i* = 1, 2, …, *n* are independent, the sample paths of Levy process are almost surely right continuous with left limits (*càdlàg*)^27^,^26^. A scalar Lévy motion is characterized by linear coefficient *b*, a diffusion parameter is Brownian motion with covariance *Q*, and a nonnegative Borel measure *ν* is called Lévy jump measure of the Lévy process, defined on 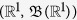 and concentrated on 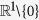, which satisfies


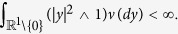


By the Lévy-Itô decomposition^27^,^42^, a Lévy process with the generating triplet (*b*, *Q*, *ν*) has the following decomposition:


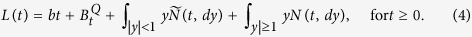


Here 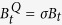, for a Brownian motion *B*_*t*_ with a covariance *Q*, such that *Q* = *σ*^2^. The process 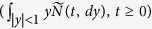 is the compensated sum of small jumps, the process 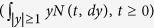 is a compound Poisson process used to describe the “large jumps”, where 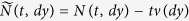. The jump measure is 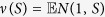, i.e., the average number of jump size in the set *S* per unit time. In present paper, we consider *α*-stable Lévy motion with a triplet (0, 0, *ν*), i.e., a pure jump motion.

The distribution for a stable Lévy random variable *L* is denoted as *S*_*α*_(*δ*, *β*, *γ*). Usually, *α* is called the Lévy motion index (stability index), *δ* is the scale parameter, *β* is the skewness parameter, and *γ* is the shift parameter. For 0 < *α* < 2, the stable Lévy random variable *L* has the following tail estimate^43^.


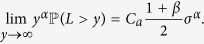


Here *C*_*α*_ is a positive constant. This estimate indicate that stable Lévy random variable *L* has “heavy tail”, as the tail estimate decays polynomially.

The symbol *S*_*α*_(*δ*, *β*, *γ*) refers to the distribution function for a stable random variable. Such as the normal distribution with 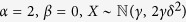, a symmetric *α*-stable Lévy distribution 
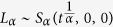
, *α* ∈ (0, 2), the jump measure is defined^43^,^42^:


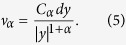


For 0 < *α* < 1, the *α*-stable Lévy motion has large jumps with lower jump probability, while it has smaller jumps with higher jump frequencies for value of *α* close to 2.

### Mean first exit time

We define the first exit time from a low concentration region *D* as follows^26^


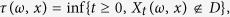


and the mean exit time is denoted as 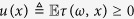. It is the mean residence time in the low concentration region before exiting to the high concentration state. By the Dynkin formula for Markov process, we obtain that the mean exit time *u*(*x*) satisfies the following determine differential-integral equation with an exterior boundary condition


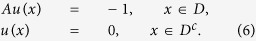


Here


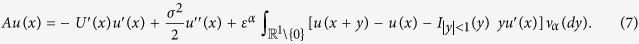


Here *D*^*c*^ is the complement set of *D* in 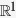. The generator *A* of the solution trajectory is defined as 
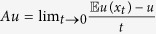
, for every 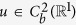.

### First escape probability

It is used to characterize the probability of the TF-A monomer concentration *X*_*t*_, first escape to the high concentration state *E* from the low concentration region *D*, denoted by *p*(*x*)^26^, that is


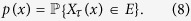


The escape probability *p*(*x*) solves the following differential-integral equation with Balayage-Dirichlet exterior boundary conditon


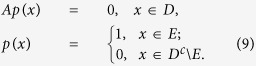


Here *A* is a generator defined in equation (7). Note that the 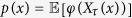 is a harmonic function respect to *X*_*t*_, which is the unique solution to the Balayage-Dirichlet problem.

### Numerical simulation

An efficient and accurate numerical finite difference scheme is developed and validated for computing the MFET and the FEP from the governing differential-integral equation by Gao *et al.*^37^. This scheme is revised for our model in *x* ∈ (*a*, *b*), by a scalar conversion 
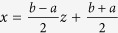
 for *z* ∈ (−1, 1), and 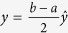, the integral of term (*I*_{|*y|*<*δ*}_*yu*′(*x*)) is zero in the symmetric *α*-stable Lévy motion. If we let 
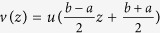
, the equation (7) of generator *A* can be discretized as following


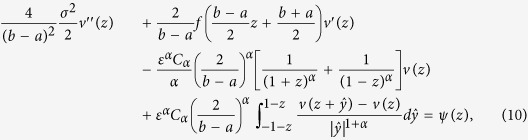


where the integral in the equation is taken as the Cauchy principal value integral. Where *ψ*(*z*) = −1or 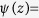 
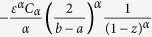
 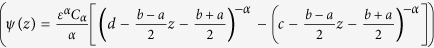 corresponds to boundary condition of the MET (Eq. 6) or the FEP (Eq. 9) in Scenario 1 (Scenario 2), respectively.

## Additional Information

**How to cite this article**: Zheng, Y. *et al.* Transitions in a genetic transcriptional regulatory system under Lévy motion. *Sci. Rep.* **6**, 29274; doi: 10.1038/srep29274 (2016).
